# Ultrastructural Characteristics of Rat Hepatic Oval Cells and Their Intercellular Contacts in the Model of Biliary Fibrosis: New Insights into Experimental Liver Fibrogenesis

**DOI:** 10.1155/2017/2721547

**Published:** 2017-07-09

**Authors:** Joanna Maria Lotowska, Maria Elzbieta Sobaniec-Lotowska, Dariusz Marek Lebensztejn, Urszula Daniluk, Piotr Sobaniec, Krzysztof Sendrowski, Jaroslaw Daniluk, Joanna Reszec, Wojciech Debek

**Affiliations:** ^1^Department of Medical Pathomorphology, Medical University of Bialystok, Bialystok, Poland; ^2^Department of Pediatrics, Gastroenterology and Allergology, Medical University of Bialystok, Bialystok, Poland; ^3^Department of Pediatric Neurology and Rehabilitation, Medical University of Bialystok, Bialystok, Poland; ^4^Department of Gastroenterology and Internal Medicine, Medical University of Bialystok, Bialystok, Poland; ^5^Department of Pediatric Surgery, Medical University of Bialystok, Bialystok, Poland

## Abstract

**Purpose:**

Recently, it has been emphasized that hepatic progenitor/oval cells (HPCs) are significantly involved in liver fibrogenesis. We evaluated the multipotential population of HPCs by transmission electron microscope (TEM), including relations with adherent hepatic nonparenchymal cells (NPCs) in rats with biliary fibrosis induced by bile duct ligation (BDL).

**Methods:**

The study used 6-week-old Wistar Crl: WI(Han) rats after BDL for 1, 6, and 8 weeks.

**Results:**

Current ultrastructural analysis showed considerable proliferation of HPCs in experimental intensive biliary fibrosis. HPCs formed proliferating bile ductules and were scattered in periportal connective tissue. We distinguished 4 main types of HPCs: 0, I, II (bile duct-like cells; most common), and III (hepatocyte-like cells). We observed, very seldom presented in literature, cellular interactions between HPCs and adjacent NPCs, especially commonly found transitional hepatic stellate cells (T-HSCs) and Kupffer cells/macrophages. We showed the phenomenon of penetration of the basement membrane of proliferating bile ductules by cytoplasmic processes sent by T-HSCs and the formation of direct cell-cell contact with ductular epithelial cells related to HPCs.

**Conclusions:**

HPC proliferation induced by BDL evidently promotes portal fibrogenesis. Better understanding of the complex cellular interactions between HPCs and adjacent NPCs, especially T-HSCs, may help develop antifibrotic therapies in the future.

## 1. Introduction

Hepatic fibrosis and end-stage liver cirrhosis are still a serious worldwide healthcare problem [1–4]. Unfortunately, the precise mechanisms underlying liver fibrogenesis are not well understood. Hepatic fibrogenesis has a dynamic nature and is a complex process that involves marked accumulation of the extracellular matrix (ECM) components, activation of cells capable of producing matrix materials, cytokine release, and tissue remodeling regulated by matrix metalloproteinases and their inhibitors [1, 4–9]. It is assumed that hepatic stellate cells (HSCs) and myofibroblasts play a crucial role in the process of liver fibrosis, including extensive remodeling of ECM components and deposition of fibrillary collagen types I and III. It is frequently emphasized that activated HSCs/myofibroblasts, especially the transitional form of HSCs (T-HSCs), defined as the main fibrogenic cell type in the liver, constitute a major source of fibril-forming ECM in this organ [5, 6, 9–13].

Recently, it has been emphasized that not only activated HSCs/myofibroblasts but also stem cells within the liver, that is, hepatic progenitor cells, in rodents collectively termed oval cells, or activated cholangiocytes, that is, epithelial cells of small ductular proliferation related to progenitor cells, are involved in the experimental models of hepatic fibrosis, especially caused by bile duct ligation (BDL) [13–18]. They may contribute to the explanation of hepatic fibrogenesis in obstructive cholangiopathy in humans [13, 17–19].

It is worthy of note that hepatic progenitor/oval cells (HPCs) constituting a heterogenic cell population and in healthy liver accounting for only 1%–3% of normal liver cell pool, located mainly in the portal and periportal areas, are not morphologically easy to recognize. This is associated with a very small size of hepatic oval cells which cannot be detected using routine histological diagnostics. Their identification with immunohistochemical markers (c-kit, CD34, Ov6, CK7, CK19, chromogranin A, CD56) also remains particularly difficult as these cells are very few in number and most of the markers are not specific [20–22]. Moreover, the molecular identification of HPCs has been hampered by the lack of truly specific markers [23].

Thus, transmission electron microscopic (TEM) analysis is thought to be the most reliable and valuable, although extremely laborious, method of morphological identification of HPCs and its various cell types [14, 22, 24–26].

In the research into the mechanisms of cholestatic liver diseases, portal fibrosis seems to be induced by ductular reaction [14, 19, 27, 28]. It has been shown that activated cholangiocytes (ductal epithelium) related to HPCs can express fibrogenic growth factors, such as transforming growth factor-*β* (TGF*β*1, TGF*β*2) [8, 15, 27–29], connective tissue growth factor (CTGF) [8], monocyte chemoattractant protein-1 (MPC-1) [11, 30], platelet-derived growth factor- (PDGF-) BB [31], and de novo expressed profibrogenic integrin *α*v*β*6 that attracts and activates hepatic stellate cells [32], leading to the accumulation of ECM proteins and collagen deposition [5, 13, 15, 29].

Although in the past two decades impressive progress has been made in our understanding of the principles that underlie the dynamic nature of liver fibrosis, especially the biochemical ones, the morphogenesis and development of this pathology at the ultrastructural level, mainly cellular interactions in progressive fibrosis, are still unknown. This particularly refers to cells deriving from epithelial-to-mesenchymal transition [12, 13, 19, 28, 33, 34], defined as the profibrogenic crosstalk between activated cholangiocytes that are related to HPCs and activated HSCs/myofibroblasts [13].

Therefore, the current study objective was ultrastructural analysis of the multipotential population of HPCs, including their relations with adherent hepatic nonparenchymal cells (NPCs), especially HSCs/myofibroblasts in the course of periportal fibroductular reaction in the model of biliary fibrosis in young rats caused by bile duct ligation. We were mainly concerned with early cellular events in the liver during the proliferative response induced by BDL. We do hope that the application of the electron microscopic investigations apart from the identification of particular types of cells among the population of HPCs will allow a comprehensive insight into their interactions with the surrounding NPCs, especially in the context of hepatic fibrogenesis. The current research into experimental hepatic fibrosis was greatly inspired by an interesting morphological-clinical study conducted by Clouston et al. [19] on the mechanisms for intensive fibrosis in patients with chronic hepatitis, in which they demonstrated strong correlation between portal fibrosis and periportal ductular reaction with expansion of hepatic progenitor cells.

The study is a continuation of our morphological and clinical research, with a focus on the ultrastructural analysis of the role of hepatic progenitor/oval cells in fibrogenesis in various chronic liver diseases in pediatric patients [24–26], and experimental investigation of the fibroductular reaction in the same model of liver fibrosis [35].

## 2. Material and Methods

### 2.1. Animal Experimental Model

Six-week-old male Wistar Crl: WI(Han) rats were submitted to common bile duct ligation (group I) in inhalation anesthesia with a mixture of 2% isoflurane (E-Z Anesthesia, WPI, Sarasota, FL, USA) and oxygen (surgical procedure concerning BDL according to Lubel et al. [36] in the Center of Experimental Medicine of the Medical University of Bialystok). After surgery, the rats were randomly divided into three subgroups (10 animals in each subgroup) and killed after 1, 6, and 8 weeks following surgical BDL-induced cholestasis. There were two comparative groups (6 rats in each) submitted to the same anesthetic procedures as in the study subgroups: one subgroup was anesthetized with isoflurane by inhalation (group II) and the other only with ketamine intramuscularly (group III); the material was collected 10 min after inhaled anesthesia with isoflurane or ketamine. Then, the animals were killed by making a cut through the cervical spine. In order to demonstrate the efficacy of the applied model of liver fibrosis, the electron microscopic investigations were preceded by histopathological assessment of liver specimens collected from all the animals. Bile ductule proliferation and inflammatory infiltrate were studied by Mayer's hematoxylin and eosin stain, whereas liver fibrosis was determined by the panel of stains for elements of fibrous tissue; fibrosis stage and inflammation grade were assessed by the Batts and Ludwig numerical scoring system [37]. Details of the methods and histopathological findings were presented in our previous report [35]. It is worthy of note that in the histopathological assessment we observed a progressive periportal fibroductular reaction that caused rebuilding of the organ architecture. In weeks 6 and 8, there was an intense disorganization of the lobules with the formation of regenerative nodules and cirrhosis (Supplementary Figures 1a and 1b available online at https://doi.org/10.1155/2017/2721547).

Material for ultrastructural analysis was obtained from 6 study animals from each research group and 3 animals from each analogous comparative group.

The study was approved by the Ethical Committee of the Medical University of Bialystok.

### 2.2. Liver Tissue Processing for Electron Microscopy

For TEM, fresh small tissue blocks (1 mm^3^ volume) from liver bioptates were fixed in the modified Karnovsky's fixative (in a solution containing 2.5% glutaraldehyde and 2% paraformaldehyde in 0.1 M cacodylate buffer, pH 7.4, at room temperature). Subsequently, the specimens were postfixed in 2% osmium tetroxide (OsO_4_) in 0.1 M cacodylate buffer, pH 7.4, for 1 h. Then, the material was dehydrated through a graded series of ethanols and propylene oxide, embedded in Epon 812 and sectioned on a Reichert ultramicrotome (Reichert Ultracut S) to obtain semithin sections. Next, the sections were stained with 1% methylene blue in 1% sodium borate and preliminarily examined under a light microscope to select Epon blocks. The blocks were then used to prepare ultrathin sections (60–70 nm thick), which were placed on grids, contrasted with uranyl acetate and lead citrate, examined using an Opton EM 900 transmission electron microscope (Zeiss, Oberkochen, Germany), and photographed with TRS camera (CCD camera for TEM 2K inside). This processing procedure had been used in our earlier ultrastructural investigations of liver in children [25, 26, 38].

## 3. Results

### 3.1. Ultrastructure of HPC Population

**Figure 1:**
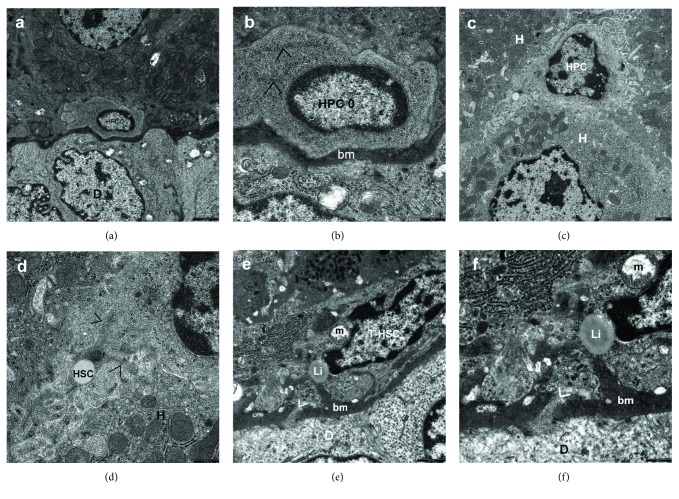
(a–f). Electron micrographs showing the periportal area of the hepatic lobule 1 week after inducing BDL in rats. (a) A very primitive-looking HPC—the periductular type 0 cell (HPC 0), corresponded to the so-called nondescript cell, located outside the basement membrane of the bile ductule. The HPC does not show any sign of differentiation or penetration of the basement membrane. The cell is very small in size, oval in shape, has scanty cytoplasm, high nucleus/cytoplasm ratio, and undifferentiated cytoplasmic and surface structures. It contains an oval nucleus in which heterochromatin is seen in the nucleoplasm with peripheral condensation. D: ductular epithelial cell. Scale bar, 2.5 *μ*m, original magnification ×4400. (b) At higher magnification, the cytoplasm of the HPC 0 is seen to contain contours of single tonofilaments (>); the basement membrane (bm) of the bile ductule. Scale bar, 0.5 *μ*m, original magnification ×20000. (c) The view of HPC differentiating towards a bile duct-like cell, pushed in the space between hepatocytes (H) with a large number of microvilli directed towards the intercellular space. The oval cell exhibits the minimum differentiated cytoplasmic structures containing numerous free ribosomes and delicate plasma membrane filapodia adhering to the microvilli. Scale bar, 1 *μ*m, original magnification ×7000. (d) Higher magnification well demonstrates the picture of cell interaction between HPC and the activated hepatic stellate cell (HSC) present at a very close distance. Processes (>) of fine HSC containing a single lipid droplet adhere tightly to the slightly folded cell membrane of the HPC. Scale bar, 0.5 *μ*m, original magnification ×20000. (e) The view of elongated transitional hepatic stellate cell (T-HSC) located outside the basement membrane (bm) of the neoformed bile ductule and intimately associated with it. The T-HSC lost a considerable part of lipid material and contains only a single tiny lipid droplet (Li). On the other hand, it has an abundance of granular endoplasmic reticulum channels. The cell nucleus is elongated, and its heterochromatin is seen as clumps of a varied size dispersed in the nucleoplasm with peripheral condensation. In the vicinity of the nucleus, markedly swollen mitochondrium (m) is visible. The basement membrane extended along the biliary ductule shows a rupture (>), with the appearance of a process directed by the T-HSC. Scale bar, 1 *μ*m, original magnification ×12000. (f) A higher magnification clearly demonstrates basement membrane penetration by the cytoplasmic process of T-HSC, causing its focal discontinuity and formation of a direct contact (>) with the ductular epithelial cell (D). Scale bar, 0.5 *μ*m, original magnification ×20000.

**Figure 2:**
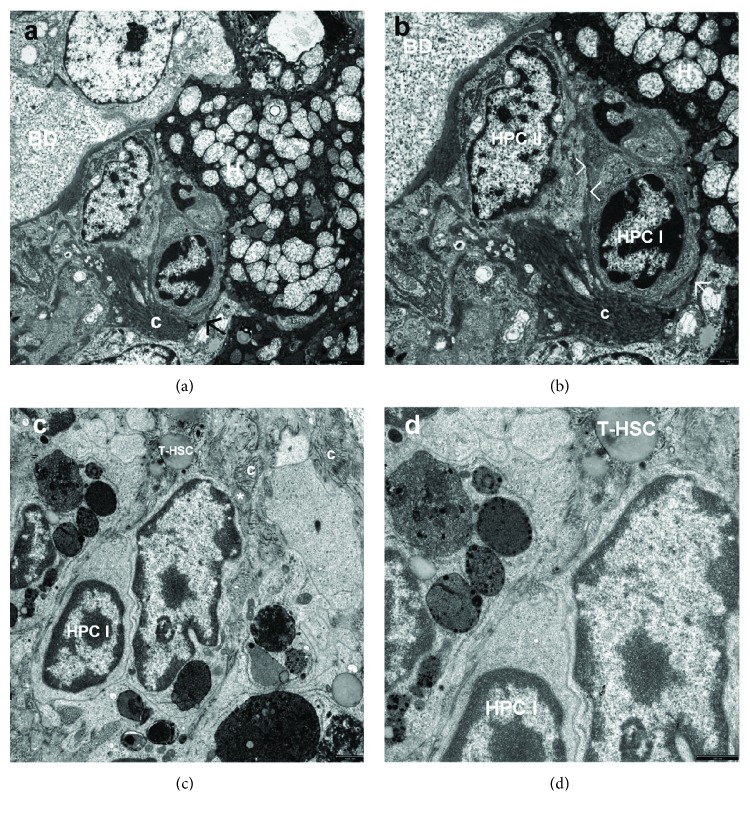
(a–d) Electron micrographs showing the periportal area of the hepatic lobule 6 weeks after inducing BDL in rats. (a) The picture of the neoforming oval cell ductule. Two HPCs (HPC I, HPC II) form the growing bile ductule in a close vicinity of the limiting plate of the hepatic lobule. These two cells are surrounded mostly by the basement membrane (→) that terminates on the periportal hepatocyte (H) showing severe mitochondrial degeneration. The basement membrane of the type II HPC is not discernible, and it seems that the cell is in contact with the collagen fiber. The HPC I (the ductal blast-like cell) adheres tightly to the adjacent cells, by tight intercellular junctions to the type II HPC (the bile duct-like cell), and to the degenerated hepatocyte. A thick bundle of collagen fibers (c) surrounds a neoforming ductular structure; in places, the bundle almost penetrates the structure. In the vicinity, a fragment of the already formed bile ductule (BD). Scale bar, 2.5 *μ*m, original magnification ×4400. (b) Higher magnification shows tight intercellular junctions (>) between two types of HPCs of the neoforming bile ductule. Scale bar, 1 *μ*m, original magnification ×12000. (c) The picture of undifferentiated HPC I—very primitive-looking cell with tightly enclosing Kupffer cells/macrophages; a process of T-HSC adheres to the upper pole of HPC I; at a short distance, a very fine degenerated HSC (^∗^) enclosed by collagen fibers (c). Scale bar, 1 *μ*m, original magnification ×12000. (d) Higher magnification well demonstrates contact HPC I with T-HSC and with Kupffer cells. Scale bar, 0.5 *μ*m, original magnification ×20000.

**Figure 3:**
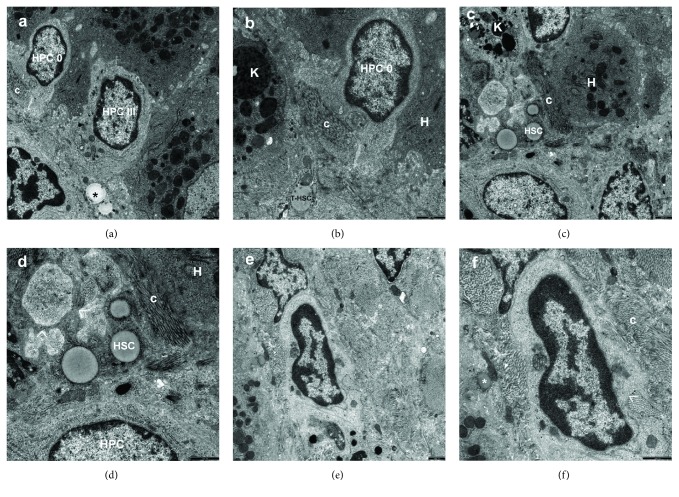
(a–f) Electron micrographs showing the periportal area (a, b) and the regenerative nodule periphery (c–f) 8 weeks after inducing BDL in rats. (a) A view of two HPCs (HPC 0, HPC III) pushed in a necrotizing hepatocyte located at the limiting plate of the lobule. In the larger cell, that is, hepatocyte-like cell (HPC III), more numerous cytoplasmic structures, mainly mitochondria and granular endoplasmic reticulum channels (in the lower cell pole), can be seen within the electron-light cytoplasm as compared to the adjacent much more primitive oval cell (HPC 0). These are mainly mitochondria and granular endoplasmic reticulum channels (in the bottom part of the HPC III). Both HPCs show very high nucleus/cytoplasm ratio and marginal location of heterochromatin. In the vicinity, fragment of the activated hepatic stellate cell (^∗^); a bundle of collagen fibers (c). Scale bar, 1 *μ*m, original magnification ×7000. (b) Higher magnification of a very primitive-looking HPC (HPC 0), morphologically corresponding to “type 0 cell,” surrounded by fragment of necrotized hepatocyte (H). A thick bundle of collagen fibers (c) adheres directly to HPC; focally, the bundle pushes in the cell. In a close vicinity of the cell, fragments of activated nonparenchymal liver cells—Kupffer cell/macrophage (K) abundant in phagolysosomes and the transitional hepatic stellate cell (T-HSC). Scale bar, 1 *μ*m, original magnification ×12000. (c) The body of an activated hepatic stellate cell (HSC) tightly adheres to a fragment of one out of two HPCs lying aside (bottom part of the electronogram). Cytoplasmic processes of the HSC directly communicate with a fragment of markedly activated Kupffer cell/macrophage (K), abundant in phagolysosomes. A thick bundle of collagen fibers (c) adheres tightly to the HSC; a round fragment of degenerating hepatocyte (H), detaching from the injured limiting plate of the lobule is visible. Scale bar, 1 *μ*m, original magnification ×7000. (d) Higher magnification shows adherence of activated HSC to HPC and displays cytoplasmic processes (>) arising from the HSC. Scale bar, 1 *μ*m, original magnification ×12000. (e) The view of HPC, differentiating towards a bile duct-like cell, situated in the center of the intensive fibrotic focus. The cell has a definitely high nucleus/cytoplasm ratio and shows the presence of few primitive cellular organelles, mainly mitochondria. Scale bar, 1 *μ*m, original magnification ×7000. (f) Higher magnification shows delicately contoured plasma membrane filapodia in the lower part of the cell. The cell is pressed directly from the outside by thick bundles of collagen fibers (c), focally pushing in it (>); in the vicinity, a degenerated HSC (^∗^), containing a single fine lipid droplet, is visible. Scale bar, 1 *μ*m, original magnification ×12000.

The analysis of liver bioptates from young Wistar Crl: WI(Han) rats subjected to BDL conducted at the electron microscopic level revealed that biliary obstruction caused bile duct cell hyperplasia throughout the liver, with an increased number of HPCs (Figures 1–3), and fibrogenesis. Hepatic fibrosis increased significantly with the disease severity from mild to severe (Figures 2(a), 2(b), 2(c), 3(b), 3(c), 3(d), 3(e), and 3(f)).

In all comparative animals, liver parenchyma had normal structure and sporadically contained single oval cells, mainly type 0 or basal, within the portal and periportal spaces (Supplementary Figure 2 and Figure 3). Their ultrastructural picture corresponded to the type of cells described below.

The expansion of HPCs encountered in the animals subjected to BDL occurred in two morphological forms: periportal ductular reaction form and intraparenchymal isolated hepatic progenitor cell form.

The current ultrastructural study allowed the identification of four types of HPCs associated with biliary fibrosis: type 0 cells; type I cells—undifferentiated HPCs; HPCs with signs of biliary differentiation, defined as type II cells, that is, the bile duct-like cells; and HPCs with ultrastructural features of differentiation towards hepatocytes—type III cells, that is, the hepatocyte-like cells.

Type 0 cells were the most primitive-looking cell type in the liver, constituting the small blast-like cell compartment. In our opinion, they corresponded to the so-called “nondescript” periductal and periductular cells. The type “0 cells,” as well as type “I cells,” of HPCs were seen in the periportal area, where they constituted the first proliferating compartment of HPCs. They were located outside the basement membrane of growing or fully mature bile ductules or at a short distance from them and contained no electron microscopic features to identify them (Figures 1(a), 1(b), 3(a), and 3(b)). We did not observe any morphological sign indicating that these very primitive periductular cells penetrated the basement membrane during the process of biliary system differentiation (Figures 1(a) and 1(b)).

Type I cells, also very primitive-looking cells, referred to as “basal cells” inside the bile ducts/ductules (syn. undifferentiated HPCs; small, intraepithelial cells), were found in all rats of the BDL groups. These cells were mainly located in proliferating bile ductules, which were composed of at least 2 cells (Figures 2(a) and 2(b)). They were also seen as single scattered cells in the fibrous septa and at the periphery of regenerative nodules (Figures 3(e) and 3(f)). This type of HPCs was characterized by small size (from 3.0 to 5.0 *μ*m), oval or round shape, scanty cytoplasm with a high nucleus/cytoplasm ratio and minimally differentiated cytoplasmic and surface structures. They had relatively few micro-organelles and rare tonofilaments, and their cell membrane formed tight junctions or desmosome-like junctions with adjacent cells (Figures 2(a) and 2(b)). The cells contained an oval, electron dense nucleus in which heterochromatin was seen as small clumps dispersed in the nucleoplasm with peripheral condensation (Figures 2(a), 2(b), 2(c), and 2(d)). Sometimes, a distinct nucleolus was seen within the nucleus (Figure 2(c)). Within some segments of the biliary system, some of the basal cells increased in size (to approximately 10 *μ*m) and showed higher numbers of cytoplasmic organelles and plasma membrane filapodia. However, the polarized phenotype of bile ductular epithelial cells did not develop.

Type II cells—bile duct-like cells, HPCs differentiating towards bile duct epithelial cell line—were most common among all the subtypes of oval cells observed in our experimental model of secondary fibrosis. They were located within the smaller branches of the biliary tree, including the canals of Hering, or noted as discretely scattered cells pushed in the space between hepatocytes (Figures 1(c) and 1(d)) and/or at the edge of regenerating nodules (Figures 3(e) and 3(f)), or present within the fibrous septa. The bile duct-like cells, always with undifferentiated HPCs, formed ductular structures (with a central lumen) that were elongated and tortuous extensions of the preexisting canals of Hering (Figures 2(a) and 2(b)). These cells had few organelles (Figures 1(c), 1(d), 2(a), 2(b), 3(e), and 3(f)) and formed lateral membrane interdigitations with adjacent cells (Figures 1(c), 1(d), and 2(b)). They were sometimes polarized, with microvillus extensions at the apex and basement membrane at the base.

Type III cells—hepatocyte-like cells, HPCs with ultrastructural features of differentiation towards hepatocytes—were least common among the four subtypes of oval cells. They showed different degrees of hepatocytic differentiation. These cells were located in hepatic cords and showed a tendency to form bile canaliculi. They exhibited more cytoplasm and a larger number of organelles than the undifferentiated HPCs, had tight junctions with other adjacent cells, and contained relatively distinct mitochondria and granular endoplasmic reticulum (Figure 3(a)). Some had lysosomes and poorly developed Golgi apparatus.

It is worthy of note that HPCs were found mainly in the proliferating bile ductules, defined as oval cell ductules of the biliary tree, where they formed periportal ductular structures representing an extension of the canals of Hering (Figures 2(a) and 2(b)). We also observed the occurrence of isolated HPCs beyond the growing biliary tree (Figures 1(a), 1(b), 1(c), and 1(d)), especially in the periportal connective tissue present in the already developed or being formed fibrotic foci and on the margin of regenerative nodules (Figures 2(c), 3(a), 3(b), 3(c), 3(d), 3(e), and 3(f)). Sometimes, the oval cells were situated between preexistent fully mature hepatocytes (Figures 3(a) and 3(b)). They were seen as single scattered cells (Figures 3(e) and 3(f)) as well as closely lying 2-3 cells in the fibrous septa and at the periphery of regenerating nodules (Figures 3(a) and 3(c)). The oval cell ductules were always surrounded by a continuous basement membrane that terminated on the hepatocyte of the limiting plate. The basement membrane had an open end plugged by a hepatocyte lying close to it (Figures 2(a) and 2(b)). However, there were segments along proliferating bile ductules, in which no structured basement membrane could be seen at the electron microscopic level.

### 3.2. The Intercellular Contacts between HPCs and Adjacent NPCs

In all experimental groups, along with the onset and development of fibrogenesis, the populations of the activated hepatic stellate cells, including transitional HSCs, were frequently observed near the proliferating bile ductules or very close to isolated HPCs at the edge of regenerative nodules and in the fibrous septa (Figures 1(c), 1(d), 1(e), 1(f), 2(c), 2(d), 3(a), 3(b), 3(c), and 3(d)). After 6 and 8 weeks of biliary obstruction due to BDL, apart from activated HSCs, degenerated HSCs were sometimes found in the foci of fibrosis, close to HPCs (Figures 2(c) and 3(f)). The T-HSCs, being morphologically very significant in the process of fibrogenesis, appeared as elongated cells that lost a considerable part or almost all lipid materials (Figures 1(e) and 1(f)) and contained well developed, sometimes dilated canals of granular endoplasmic reticulum (Figures 1(e), 1(f), and 3(b)) and Golgi apparatus. Interestingly, T-HSCs and their processes observed outside the basement membrane of growing and mature bile ductules showed very intimate connection with the biliary tree. Sometimes, although seldom, the processes of T-HSCs penetrated the basement membrane, caused its focal discontinuity, and formed a direct cell-cell contact with the HPCs that showed biliary epithelial cell differentiation or with the already mature ductular epithelial cells (Figures 1(e) and 1(f)).

It should be noted that in the respective experimental groups, activated Kupffer cells/macrophages (KCs/MPs) were frequently observed adhering directly to HPCs (Figures 2(c) and 2(d)) or in their close vicinity (Figures 3(b) and 3(c)).

## 4. Discussion

It is assumed that the BDL rat model is the prototype animal model for “typical” cholangiocyte proliferation [39]. It results in a reproducible portal tract fibrosis, resembling human chronic biliary obstruction, over a short period of time (4–6 weeks in rats, 2-3 weeks in mice) [13, 15, 17, 28, 39, 40]. Our earlier histopathological study on experimental biliary atresia due to BDL in young Wistar Crl: WI(Han) rats indicated a spontaneous and progressive character of fibroductular reaction contributing to periportal fibrogenesis [35].

However, the current electron microscopic analysis conducted in the same BDL-treated rats showed, especially after 6 and 8 weeks of the experiment, a striking relationship between increasing hepatic fibrosis and hepatic progenitor/oval cell expansion associated with periportal ductular reaction. HPCs were mainly located in proliferating bile ductules and also occurred as intraparenchymal isolated oval cells in periportal connective tissue or scattered in the fibrous septa and at the periphery of regenerative nodules. Interestingly, in the close vicinity of HPCs, NPCs, especially activated HSCs/myofibroblasts and activated KCs/MPs, were commonly found. Our study, based on the classical experimental model of secondary biliary fibrosis, distinguished 4 main types of HPCs: “type 0 cells”; “type I cells”—undifferentiated HPCs referred to as “basal cells”; “type II cells”—bile duct-like cells; and “type III cells”—hepatocyte-like cells—oval cells with ultrastructural features of differentiation towards biliary and hepatocyte lineages. Most frequently, we found “type II cells,” that is, cells with signs of biliary differentiation.

The current study results are consistent with the ultrastructural data reported by other authors in various experimental models of liver injury, mainly associated with atypical ductular reaction [14, 16, 18]. Our findings are also similar to those observed in a variety of human chronic liver diseases accompanied by hepatic fibrosis, especially in obstructive cholangiopathy, in the course of hepatitis B virus-positive and hepatitis C virus-positive liver cirrhosis, and in alcoholic and nonalcoholic fatty liver disease [16, 18, 41–43].

It is worthy of note that our earlier studies on the ultrastructure of the population of HPCs in certain chronic liver disorders in pediatric patients revealed a substantial contribution of these cells to the process of fibrogenesis and development of liver fibrosis in chronic type B hepatitis and in nonalcoholic steatohepatitis. Among the analyzed subpopulations of HPCs, the hepatocyte-like cells, that is, type III cells, were found to be most involved in the process of hepatic fibrosis in the abovementioned diseases in children [24–26, 38].

However, in hepatological literature, there are only few reports on experimental liver fibrogenesis with comprehensive descriptions of HPC ultrastructure, including their interactions with the population of activated HSCs/myofibroblasts. Interestingly, we found that the activated HSCs observed in the current study were always intimately associated with proliferating or mature bile ductules as well as with scattered hepatic oval cells present in the already developed or being formed fibrotic foci.

It should be noted that TEM analysis showed clearly a very rare phenomenon of penetration by cytoplasmic processes sent by activated HSCs, the basement membrane of bile ductules, and formation of direct cell-cell contact with ductular epithelial cells related to progenitor/oval cells. The ultrastructural pictures of such junctions, that is, direct cell-cell ones, have been very seldom documented in literature. Paku et al. [14] were the first to describe the ultrastructure of the junction between the activated HSC and the ductular epthelial cell. The authors cited suggest that the junction may form the structural basis for the intensive crosstalk between these two cell types [14].

Of the four types of HPCs distinguished in the current submicroscopic investigations, worthy of note are the “nondescript” periductular cells constituting an intermediary or transition population between stem cells and mature functional compartment [14, 16]. Sell [16] in his work on the morphogenesis of HPCs in human atypical ductular reactions and in the experimental model of liver injury concludes that there is a very primitive stem cell type in the liver (syn “type 0 cell”) that may differentiate directly into type I and then into type II or type III cells.

Summing up, our ultrastructural study based on the BDL rat model, resembling human chronic biliary obstruction, demonstrated a pronounced relationship between the increasing portal hepatic fibrosis and marked proliferation of HPCs, mainly type II cells with signs of biliary differentiation, within the growing intrahepatic biliary system. The HPCs, apart from their major location within the neoforming bile ductules, were also found in the form of isolated oval cells in periportal connective tissue, in the already developed or being formed fibrotic foci and at the periphery of regenerative nodules. We support the hypothesis forwarded by other authors that HPCs are derived from biliary epithelium, indicating that liver stem cells are located in the biliary system [12–14, 19, 41]. Thus, the biliary epithelial cells may presumably be the precursors of hepatic oval cells. This, however, requires further comprehensive morphological studies. In the current report, we also analyzed an interesting ultrastructural picture of intercellular contacts between HPCs and adjacent NPCs, especially between the frequently found activated HSCs and activated KCs/MPs. It is worth noting that our study documented an extremely rare phenomenon of penetration of the basement membrane of bile ductules by cytoplasmic processes sent by T-HSCs and the formation of direct cell-cell contact with ductular epithelial cells related to HPCs. The association between the severity of liver fibrosis and proliferation of oval cells is consistent with the hypothesis that HPCs could send signals to HSCs and thus increase the risk of fibrogenesis in chronic liver diseases [4].

## 5. Conclusions

The current ultrastructural assessment indicates that the expansion of HPCs, mainly bile duct-like cells, induced by BDL in young rats occurring in two morphological forms, that is, periportal ductular reaction form and intraparenchymal isolated hepatic progenitor cell form, evidently promotes portal hepatic fibrosis, probably by involvement in signaling to hepatic stellate cells. The research clearly shows that the presence of an increased number of T-HSCs very close to HPCs was a major morphological exponent of fibroductular reaction, which sheds a new light on a very complicated process of liver fibrosis. We hope that a better understanding of the complex cellular interactions observed between HPCs and the adhering NPCs, especially the activated HSCs, regulating experimental biliary fibrogenesis, might be a potential value in patients with chronic liver diseases and help to develop antifibrotic therapies in the near future.

## Supplementary Material

The information of supplementary materials are as follows: Supplemental Figure 1a, b: The histological picture of rat liver specimens collected 6 weeks after surgical BDL. Interesting is the intense ductular proliferation occurring in a radiated form towards the parenchyma of the liver lobules, forming thinner and thicker septa. a: The architecture of the liver parenchyma is prominently disturbed; delimitation of portal and periportal areas is visible; numerous regenerative nodules are seen to develop (staining H&E, original magnification x 100). b: Higher magnification well demonstrates the extension of the ductular reaction into the parenchyma and a distinct regenerative nodule being formed (staining H&E, original magnification x 200). Supplemental Figure 2: Electron micrographs showing a very primitive-looking, undifferentiated HPC (HPC type I), centrally located in the space between hepatocytes of the periportal area (in the center of electronogram) obtained from a young control rat anesthetized with isofluorane by inhalation. The HPC I is very small in size, oval in shape, has scanty electron-light cytoplasm with a definitely high nucleus/cytoplasm ratio and exhibits the minimum quantity of differentiated cytoplasmic structures; cellular cytoplasm is much brighter than the surrounding hepatocytes. The cell contains a large, oval nucleus, in which heterochromatin is seen as small clumps dispersed in the nucleoplasm with distinct peripheral condensation. Hepatocytes surrounding HPC I show a well preserved ultrastructure with large microvilli directed towards the intercellular space. Scale bar, 1 µm, original magnification x 12 000. Supplemental Figure 3: The view of a HPC, differentiating towards a bile duct-like cell, situated in the center of electron micrograph, in the space between hepatocytes in the periportal area, obtained from a young control rat anesthetized intramuscularly with ketamine. The cell is very small in size, oval in shape, has a high nucleus/cytoplasm ratio and shows the presence of single primitive cellular organelles within relatively scarce, electron-light cytoplasm; a single mitochondrion and delicately contoured plasma membrane filapodia can be seen. The nucleus contains dense heterochromatin accumulated markedly under the nuclear envelope and less abundant euchromatin.Scale bar, 1 µm, original magnification x 12 000.
