# Supplementary material for: Ultrastructural Characteristics of Rat Hepatic Oval Cells and Their Intercellular Contacts in the Model of Biliary Fibrosis: New Insights into Experimental Liver Fibrogenesis
Source: Gastroenterol Res Pract. 2017 Jul 9;2017:2721547. doi: 10.1155/2017/2721547 (PMC5523291; doi:10.1155/2017/2721547)
Supplement: Supplementary file 1 — The information of supplementary materials are as follows: Supplemental Figure 1a, b: The histological picture of rat liver specimens collected 6 weeks after surgical BDL. Interesting is the intense ductular proliferation occurring in a radiated form towards the parenchyma of the liver lobules, forming thinner and thicker septa. a: The architecture of the liver parenchyma is prominently disturbed; delimitation of portal and periportal areas is visible; numerous regenerative nodules are seen to develop (staining H&E, original magnification x 100). b: Higher magnification well demonstrates the extension of the ductular reaction into the parenchyma and a distinct regenerative nodule being formed (staining H&E, original magnification x 200). Supplemental Figure 2: Electron micrographs showing a very primitive-looking, undifferentiated HPC (HPC type I), centrally located in the space between hepatocytes of the periportal area (in the center of electronogram) obtained from a young control rat anesthetized with isofluorane by inhalation. The HPC I is very small in size, oval in shape, has scanty electron-light cytoplasm with a definitely high nucleus/cytoplasm ratio and exhibits the minimum quantity of differentiated cytoplasmic structures; cellular cytoplasm is much brighter than the surrounding hepatocytes. The cell contains a large, oval nucleus, in which heterochromatin is seen as small clumps dispersed in the nucleoplasm with distinct peripheral condensation. Hepatocytes surrounding HPC I show a well preserved ultrastructure with large microvilli directed towards the intercellular space. Scale bar, 1 µm, original magnification x 12 000. Supplemental Figure 3: The view of a HPC, differentiating towards a bile duct-like cell, situated in the center of electron micrograph, in the space between hepatocytes in the periportal area, obtained from a young control rat anesthetized intramuscularly with ketamine. The cell is very small in size, oval in shape, has a high nucleus/ [file 2721547.f1.docx]

**Supplemental Figure 1a, b**

The histological picture of rat liver specimens collected 6 weeks after surgical BDL.

Interesting is the intense ductular proliferation occurring in a radiated form towards the parenchyma of the liver lobules, forming thinner and thicker septa.

**a:** The architecture of the liver parenchyma is prominently disturbed; delimitation of portal and periportal areas is visible; numerous regenerative nodules are seen to develop (staining H&E, original magnification x 100).

**b:** Higher magnification well demonstrates the extension of the ductular reaction into the parenchyma and a distinct regenerative nodule being formed (staining H&E, original magnification x 200).


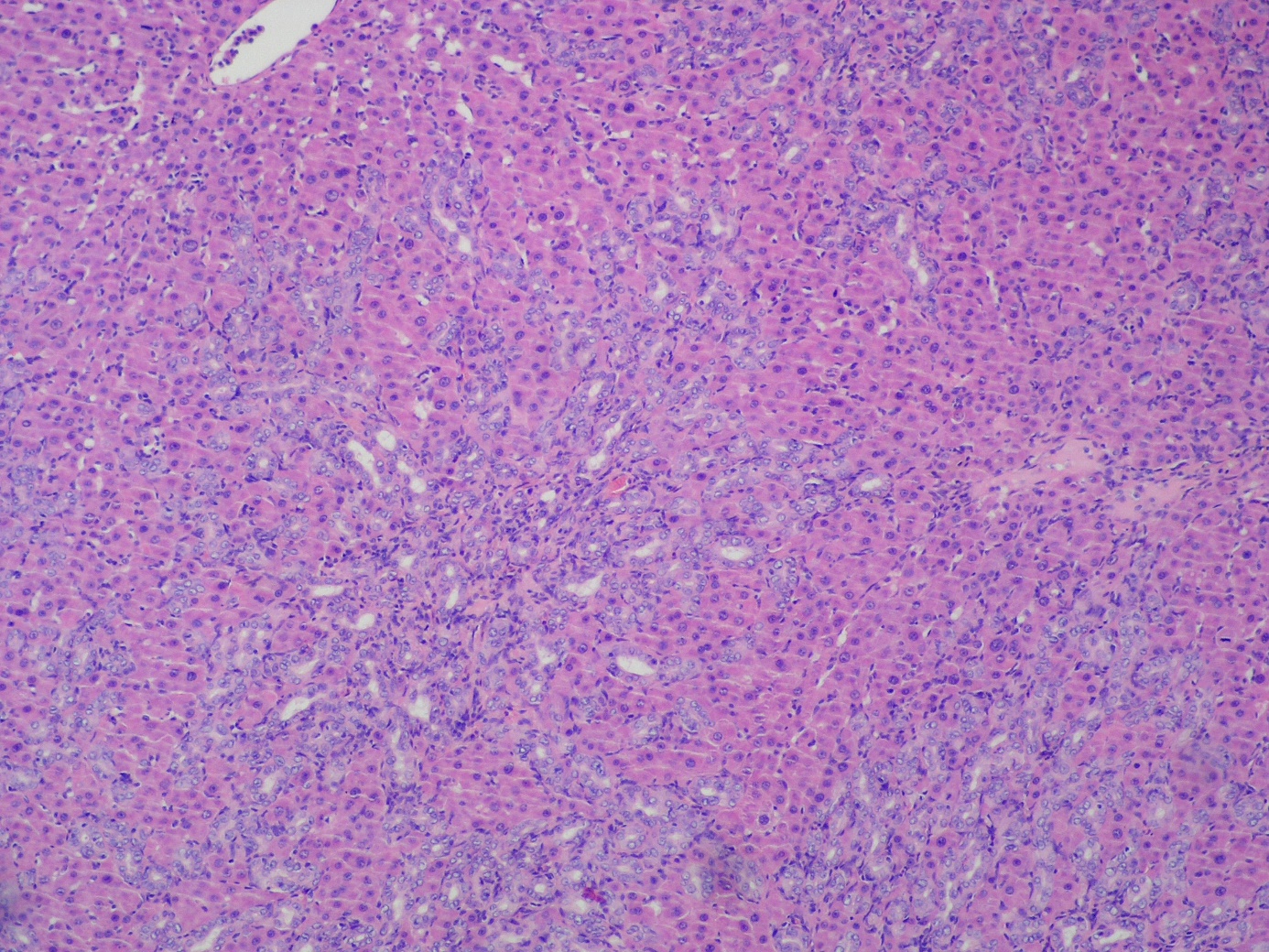


a


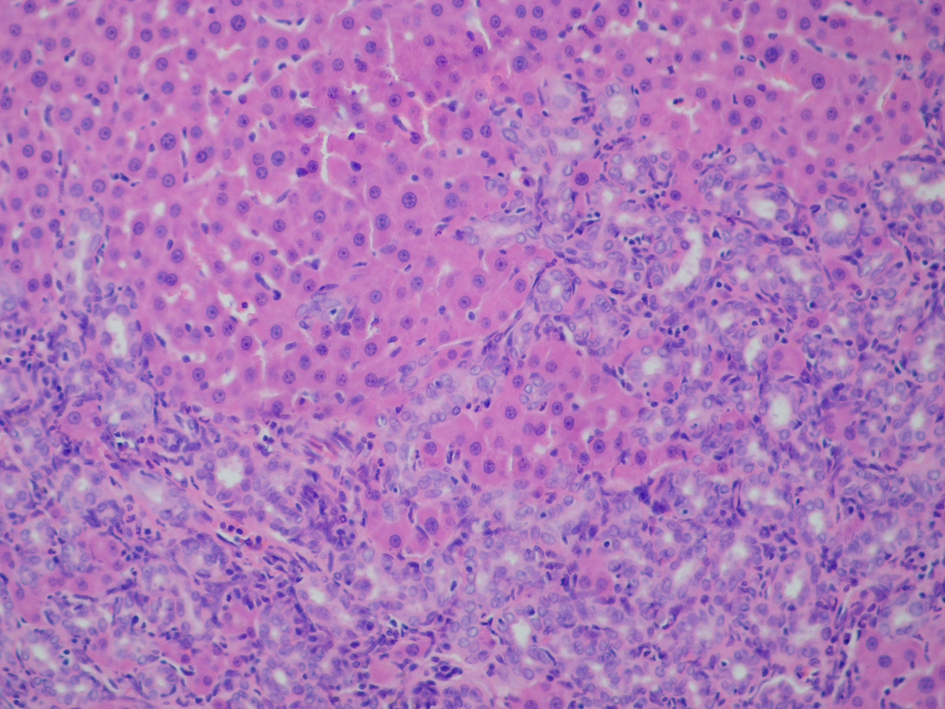


b
