# Supplementary material for: Ultrastructural Characteristics of Rat Hepatic Oval Cells and Their Intercellular Contacts in the Model of Biliary Fibrosis: New Insights into Experimental Liver Fibrogenesis
Source: Gastroenterol Res Pract. 2017 Jul 9;2017:2721547. doi: 10.1155/2017/2721547 (PMC5523291; doi:10.1155/2017/2721547)
Supplement: Supplementary file 2 [file 2721547.f2.docx]

**Supplemental Figure 2**

Electron micrographs showing a very primitive-looking, undifferentiated HPC (HPC type I), centrally located in the space between hepatocytes of the periportal area (in the center of electronogram) obtained from a young control rat anesthetized with isofluorane by inhalation.

The HPC I is very small in size, oval in shape, has scanty electron-light cytoplasm with a definitely high nucleus/cytoplasm ratio and exhibits the minimum quantity of differentiated cytoplasmic structures; cellular cytoplasm is much brighter than the surrounding hepatocytes. The cell contains a large, oval nucleus, in which heterochromatin is seen as small clumps dispersed in the nucleoplasm with distinct peripheral condensation.

Hepatocytes surrounding HPC I show a well preserved ultrastructure with large microvilli directed towards the intercellular space.

Scale bar, 1 µm, original magnification x 12 000.

**
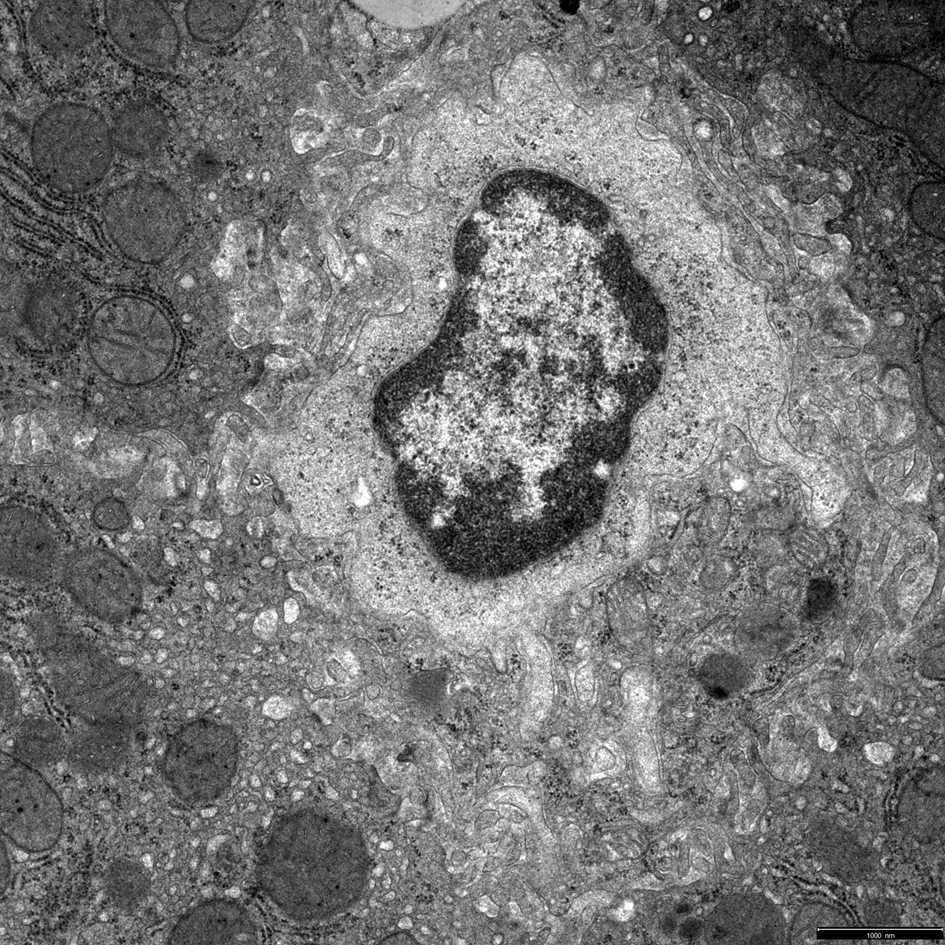
**
