# Supplementary material for: Ultrastructural Characteristics of Rat Hepatic Oval Cells and Their Intercellular Contacts in the Model of Biliary Fibrosis: New Insights into Experimental Liver Fibrogenesis
Source: Gastroenterol Res Pract. 2017 Jul 9;2017:2721547. doi: 10.1155/2017/2721547 (PMC5523291; doi:10.1155/2017/2721547)

**Supplemental Figure 3**

The view of a HPC, differentiating towards a bile duct-like cell, situated in the center of electron micrograph, in the space between hepatocytes in the periportal area, obtained from a young control rat anesthetized intramuscularly with ketamine.

The cell is very small in size, oval in shape, has a high nucleus/cytoplasm ratio and shows the presence of single primitive cellular organelles within relatively scarce, electron-light cytoplasm; a single mitochondrion and delicately contoured plasma membrane filapodia can be seen. The nucleus contains dense heterochromatin accumulated markedly under the nuclear envelope and less abundant euchromatin.

Scale bar, 1 µm, original magnification x 12 000.


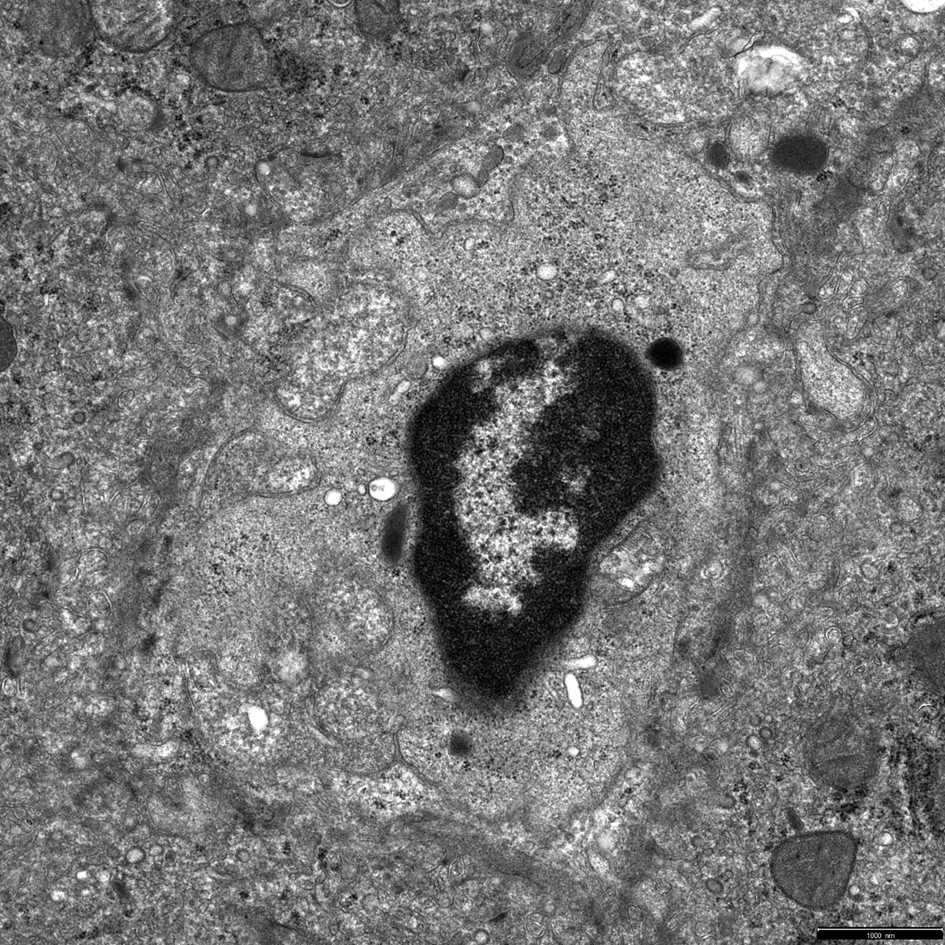

Supplement: Supplementary file 3 [file 2721547.f3.docx]
